# Seasonal changes in physiological and psychological parameters of stress in collegiate swimmers

**DOI:** 10.1038/s41598-023-37124-x

**Published:** 2023-07-07

**Authors:** Haoyan Wang, Bailey M. Theall, Kate S. Early, Cullen Vincellette, Lyle Robelot, Rick L. Sharp, Jack Marucci, Shelly Mullenix, Derek Calvert, Nathan P. Lemoine, Brain A. Irving, Guillaume Spielmann, Neil M. Johannsen

**Affiliations:** 1 College of Physical Education and Health Sciences, Zhejiang Normal University, Jinhua, China; 2 School of Kinesiology, Louisiana State University, Baton Rouge, LA USA; 3 Department of Kinesiology and Health Sciences, Columbus State University, Columbus, GA USA; 4 Department of Athletics, Louisiana State University, Baton Rouge, LA USA; 5 College of Human Sciences, Iowa State University, Ames, IA USA

**Keywords:** Health care, Signs and symptoms

## Abstract

To investigate the seasonal changes in physiological and psychological parameters of stress in collegiate swimmers. Fifteen NCAA Division I swimmers (8 men) participated in a tethered anaerobic swim test to determine physiological responses in an ecologically-relevant, graded exercise test. Wisconsin Upper Respiratory Symptom Survey (WURSS-21), Activation-Deactivation Adjective Check List (AD-ACL), Daily Analysis of Life Demands of Athletes (DALDA), and Pittsburgh Sleep Quality Index were assessed at post-season in April (V_1_), the end of off-season in June (V_2_), and pre-season in October (V_3_). The percent change was determined from V_2_–V_1_ (off-season phase), V_3_–V_2_ (pre-season phase), V_1_–V_3_ (in-season phase). Spearman’s rho correlation was used to examine associations between change in physiological and psychological outcomes. All data results showed a better swim performance occurred at V_2_. Men tended to have faster speed (*p* = 0.07) in fewer strokes (*p* = 0.10) and greater work per stroke (*p* = 0.10) at V_2_ than V_1_. Women were faster during V_2_ compared to V_1_ (*p* = 0.02) and V_3_ (*p* = 0.05). Women had fewer strokes (*p* = 0.02) and greater work per stroke (*p* = 0.01) at V_2_ compared to V_3_. Women had the lowest HR and lactate concentration at V_3_ compared to other visits (*p* < 0.05). During the in-season phase, swim speed decreased the greatest extent and stress sources and symptoms assessed by DALDA had greatest elevation (*p* < 0.05). An increased in stress sources and symptoms assessed by DALDA was associated with an increase in upper respiratory illness from WURSS-21 (*rho* = 0.44, *p* = 0.009), being less energetic (*rho* = − 0.35, *p* = 0.04) and greater tension state (*rho* = 0.49, *p* = 0.003; AD-ACL), and a decrease in swim speed (*rho* =− 0.38, *p* = 0.03). Swim performance peaked at off-season when psychological stress was at its lowest. The relationship between DALDA scores with psychological parameters and swim performance suggested physiological and psychological parameters of stress is an important aspect to avoid overtraining when approaching high swim performance.

## Introduction

Elite athletes frequently undergoing exercise strenuously, accumulating physical stress to optimize their athletic performance. This physical stress comes from training often results in a series of mental strain and emotional stress^1^. A previous study showed that athletes who experience chronic stress have a slower recovery thereby impairing future athletic performance, such as muscle mass, fitness, and training intensity during exercise^2^. Collegiate swimmers participate in highly competitive training across a variety of events, with several athletes exercising at Olympic level volumes. Depending on the time of year, elite swimmers may train at volumes up to ~ 57 km across 6–7 days per week^3^. The intense preparation for the competitive season and repetitive nature of motions can leave swimmers prone to overtraining and injuries^4^. In addition, excessive long-term training in combination with lack of recovery time can lead to fatigue or burnout in a swimmer where muscle strength, anaerobic power, and swim performance is compromised^5^. Overtraining can impact of facets of health by stimulating mood disturbances, changes in mental health and increasing susceptibility to respiratory infections^6^. In a sport where margins between first and second place are in hundredths of a second, swimmers exhibiting physiological and psychological signs and symptoms of overtraining may be unable to achieve their full potential^7^.

A well-planned, periodized training program applies alternating combinations of intensity, volume, and rest throughout a competitive season. During the competitive season, most traditional swimming programs initiate a lower training load, followed by a gradual ramp up to peak training load, and lastly a load reduction to allow for adequate recovery prior to major competitions^8^. However, swimmers may or may not be prepared for major competitions based on training volume alone as this is a gauge of training progression. Monitoring athletes for psychological variables like stress and fatigue may enhance an athlete’s readiness and sports performance by examining resilience across a season. However, the connection between these widely accepted subjective measures of well-being and objective parameters of health and performance is inconsistent. In various sports, training responses should be detected from the relationships between psychological state (i.e., mood disturbance, stress, sleep, fatigue), physiological measures (immune and inflammation markers), and athletic performance^9^.

Anaerobic power is commonly assessed to determine swimming performance and signs and symptoms of overtraining^10^. The tethered swim test is an ecologically-relevant, valid and reliable sport-specific test that measures propulsive force production in water^11^. A swimmer can perform any stroke while a cable is attached to the body with a specific workload assessing the work production per stroke, and overall power output^12^. This unique anaerobic test can help practitioners and coaches’ alike to understand swimming performance (speed, work, power output) in addition to physiological adaptations in a swim-relevant environment. To our knowledge, no previous study has attempted to characterize changes in anaerobic tethered swim performance longitudinally across a season.

Collegiate swimmers participate in a variety of training regimes including in dry-dock conditioning, daily in-water training, and competition events spanning several academic semesters. Pressure to maintain fitness in addition to the time commitment to academic may place excessive psychological stress on swimmers and potentially interfere with swim performance^13^. Therefore, the purpose of this study was to investigate the physiological and psychological parameters of stress on three occasions during an academic year (pre-season, post-season, and off-season) in collegiate swimmers. In addition, this study sought to investigate the relationships between the physiological and psychological parameters. We hypothesized that swimmers would have peak physiological adaptations and swim performance in pre-season compared to off-season and post-season, and an increased in DALDA scores would be associated with impaired swim performance and consistent with other psychological parameters.

## Methods

### Participants

Fifteen National Collegiate Athletic Association (NCAA) Division I swimmers (age: 19.9 ± 0.6 yr, height: 177.5 ± 10.4 cm, weight: 73.5 ± 11.4 kg) participated in this study, including 8 men (3 mid-distance swimmers and 5 sprinters) and 7 women swimmers (4 mid-distance swimmers and 3 sprinters). An a priori sample size calculation was not performed as this was a convenience sample size from the swim team. The study protocol was approved by Louisiana State University Institutional Review Board (IRB#3836) and all methods were carried out in accordance with the Helsinki declaration. All participants were provided written informed consent prior to any assessment.

### Study design/procedures

The study was conducted in a longitudinal design and data collection occurred at three visits: post-season, one week after NCAA championships in April (V_1_); end of off-season, in June (V_2_); and pre-season, in October (V_3_). Target swim volume was designed by coaching staff throughout the season (sprinters, V_1_: 28.6 km/wk; V_2_: 34.5 km/wk; V_3_: 34.6 km/wk; mid-distance, V_1_: 36.6 km/wk; V_2_: 34.5 km/wk; V_3_: 42.2 km/wk). The time points were chosen to optimize access to swimmers and minimizing the invasiveness to their training program. At each visit, swimmers came in during their daily training time in the afternoon. Swimmers took 15-20 min to fill out 4 subjective psychological questionnaires. Then, an anaerobic tethered swim test was assessed.

### Anaerobic tethered swim test

**Figure 1:**
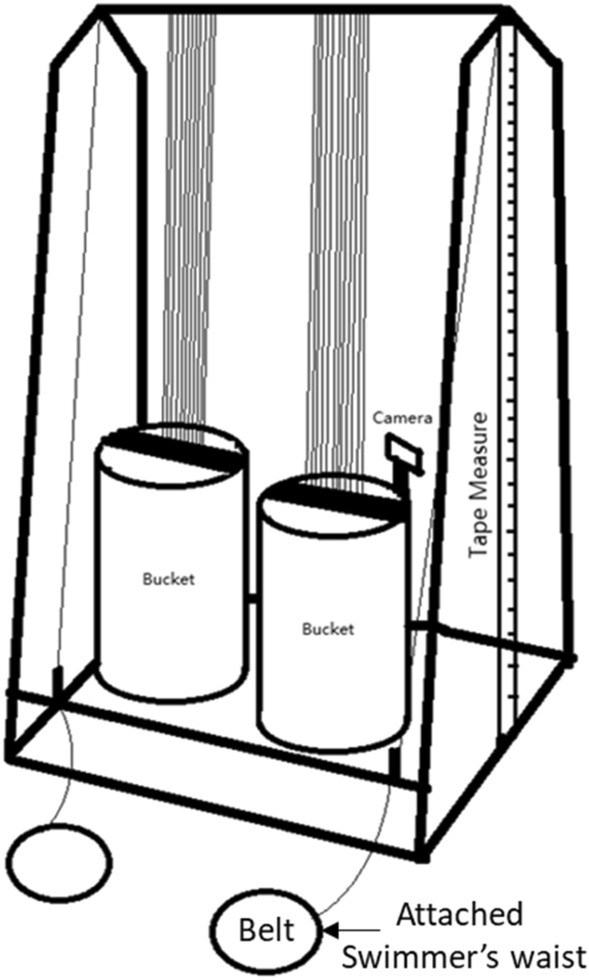
The tethered anaerobic swim test. The tethered swim test was designed for in-water power assessment consisted of a tower device and a block-and-tackle pulley weight system. A belt was worn around swimmer’s waist attaching to a pulley weight system ended with an empty bucket for adjusting resistance workload. A camera was set on the bucket facing to the tape measure recording the bucket rising distance. Anaerobic power was determined by the distance traveled per second factoring the additional resistance weight.

The tethered swim test is a graded exercise test designed to elicit a maximal in-water power assessment. The device used consisted of a tower and a block-and-tackle pulley weight system (Fig. 1). Swimmers wore a harness around the waist attached to a pulley weight system that ended with a 75L bucket. A tape measure was affixed on the side of the pulley system and a camera (GoPro Hero 4, San Mateo, CA) was set on the bucket facing the tape measures allowing for recording of the distance traveled by the bucket. The test began with a 15-min warm up followed by the incrementally-loaded freestyle swim at maximal effort. The swimmers used this pulley weight system in their daily training, and they were familiar with this test. The resistance load was designed by a team coach, and the initial resistance load was set to 18 kg for men and 9 kg for women swimmers with the weight placed in the bucket prior to each effort. Resistance was increased after each successful effort by 9 kg for men and 7 kg for women. Swimmers were encouraged to finish as fast as possible at each trial. A submaximal tethered resistance workload was selected (men at 36 kg and women at 23 kg), which ensured all swimmers are able to finish the trial for determining physiological outcomes and anaerobic power output. The swimming distance for each effort was 22.86 m and a successful effort was defined as completing the entire distance and touching the wall. After each successful loaded effort, 3 min of rest/active recovery was provided. Blood lactate concentration (La^-^; Lactate Plus Meter, Waltham MA), heart rate (HR; Garmin Forerunner 920XT), and rating of perceived exertion (RPE; Borg 6–20 scale) were measured within the first minute of each recovery period. The time for each effort was determined as push off until hands touched the opposing wall using a manual stopwatch. Anaerobic power was determined by using the distance traveled per second factoring the additional resistance weight.

### Psychological assessments

Prior to in-pool testing, psychological measurements were collected once at each visit through a series of subjective questionnaires including the Wisconsin Upper Respiratory Symptom Survey (WURSS-21), Activation-Deactivation Adjective Check List (AD-ACL), Daily Analysis of Life Demands of Athletes (DALDA), and Pittsburgh Sleep Quality Index (PSQI). Each participant was given instruction about the questionnaires and blinded to the outcome’s determination.

The WURSS-21 is a survey to determine the extent of upper respiratory infection including 4 categories composing of Likert-scale items ranging from 0 to 7 points. The categories are “How sick do you feel today” assessed by 1 item, severity of cold symptoms (10 items), functional pulmonary impairment (9 items), and sick severity change (“compared to yesterday, I feel that my cold is…”, 1 item)^14^. The sum of items (possible range 0–147) was used for analysis, with a higher score suggesting greater upper respiratory infection severity.

The AD-ACL is a validated self-assessment inventory containing 20 adjective items in 4 subscales to describe feelings or mood at the moment^15^. The 4 subscales (5 items each) are: energetic, tiredness, tension, and calmness. Participants answered each item by circling the appropriate response as “definitely feel” (+ +), “feel slightly” ( +), “cannot describe” (?), and “definitely do not feel” (no). The AD-ACL was scored out of four points per item, and 5 items averaged for each subscale (possible range 0–20). The AD-ACL score reflects the magnitude of a participant’s feeling state of activation or deactivation.

The DALDA questionnaire is a self-report inventory of life-stress and stress symptoms. The two subscales of the DALDA are sources of stress (part A) and stress symptoms (part B)^16^. There are 9 items of source of stress in part A and 25 items of stress symptoms in part B. Participants answered each item as being either “worse than normal (a)”, “normal (b)”, and “better than normal (c)” for both subscales. Only “worse than normal (a)” answers are accounted for the final score assessment (part A: 0–9 and part B: 0–25) as the questionnaire suggests increase in “a” scores indicates overreaching or overtraining for athletes^17^. The total number of “a” scores (A and B: possible range 0–34) were used for analysis.

PSQI is a self-determined questionnaire for assessing retrospective sleep quality, which is comprised of 19 items forming 7 subcategories: sleep quality, sleep latency, sleep duration, sleep efficiency, sleep disturbance, sleep medication, and daily dysfunction. Each subcategory is scored on a 3-point scale and summed for a total score (possible range 0–21). A total score of 5 or more is suggestive of poor sleep quality^18^.

### Statistical analysis

**Figure 2:**
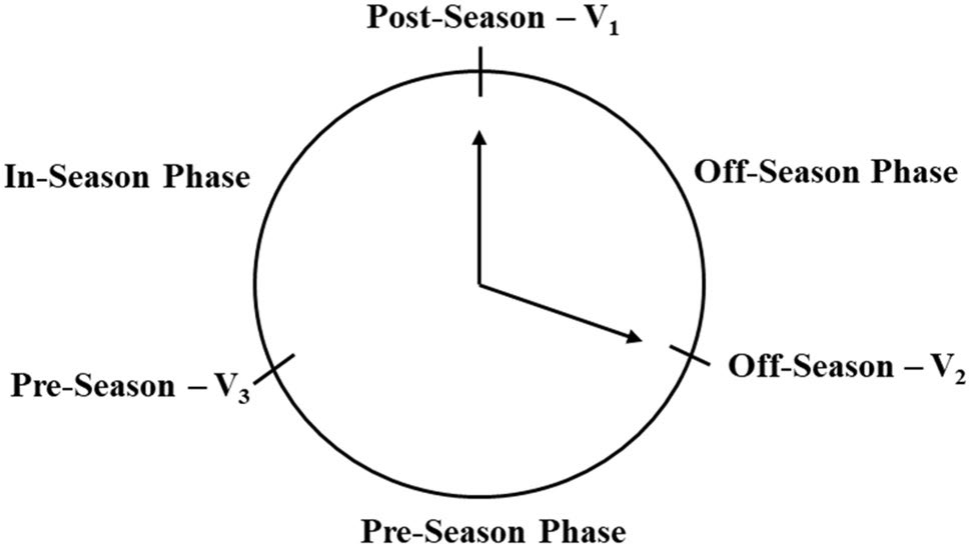
The time visit of the experiment throughout the swimming competitive season. Off-season phase: V_2_–V_1_; Pre-season phase: V_3_–V_2_; In-season phase: V_1_–V_3_.

Analyses were performed using JMPro 16 (SAS Inc., Cary, NC, USA). Data were presented as least square mean ± standard deviation (LSM ± SD). Six men and 4 women participated in all three visits, with 2 additional men and 3 additional women participating in V_1_ and V_3_ (n = 15 for V_1_ and V_3_). Physiological and psychological outcomes were assessed at all visits (V_1_, V_2_, and V_3_). There were 3 missing data due to the technical difficulties during the tethered swim tests. We did not impute missing data for the final statistical analysis. The main physiological outcomes were swim speed, power output, number of strokes, and work per stroke as well as HR, La^-^, RPE at each workload of the tethered swim test. Due to the different submaximal resistance workloads between men and women generating different mechanical work and power, the present study analyzed the physiological outcomes separately by sex. Psychological outcomes included the scores of WURSS-21, AD-ACL, DALDA, and PSQI. Mixed models, with ID as random variable and visits as the fixed effects, were used to analyze the differences of physiological and psychological parameters across visits for each sex, with Student-t post hoc pairwise comparisons between visits. A percent change was determined for outcome variables from V_2_–V_1_ (off-season–post-season: off-season phase), V_3_–V_2_ (pre-season–off-season: pre-season phase), V_1_–V_3_ (post-season–pre-season: in-season phase; Fig. 2). The change for each outcome variable was pooled from each aforementioned phase and denoted as delta (Δ) with all data combined. Spearman’s rho correlation was used to examine associations between Δ physiological and Δ psychological outcomes due to non-normal distribution of responses in the psychological variables. Statistical significance was determined at *p* < 0.05 and a trend for significance when *p* = 0.05 to *p* < 0.1.

## Results

### Physiological and psychological parameters

Table 1 shows physiological outcomes across the visits in collegiate swimmers. During off-season, men swimmers tended to have a faster average speed 0.13 m/s (*p* = 0.07) in 6 fewer strokes (*p* = 0.10) and greater work per stroke (*p* = 0.10) compared to post-season. Women were faster during off-season compared to post-season (*p* = 0.02) and pre-season (*p* = 0.05). Women tended to have fewer strokes at off-season compared to post-season (*p* = 0.07) and pre-season (*p* = 0.02), and greater work per stroke at off-season compared to pre-season (*p* = 0.01). In addition, the lowest HR and lactate concentration were observed in pre-season compared to post-season (*p* = 0.02) and off-season (*p* = 0.001) in women. As a whole, swimmers exhibited a slower speed (*p* = 0.01) and greater number of strokes (*p* = 0.02) at post-competitive season compared to off-season. RPE and peak power were not different between sex across the three visits (*p* > 0.05 for both within each sex).

**Table 1:** Physiological responses to submaximal workload in the anaerobic tethered test.

	Men swimmers			Women swimmers			All		
	V_1_ (n = 8)	V_2_ (n = 6)	V_3_ (n = 8)	V_1_ (n = 7)	V_2_ (n = 4)	V_3_ (n = 7)	V_1_ (n = 15)	V_2_ (n = 10)	V_3_ (n = 15)
Speed (m/s)	1.11 ± 0.18	1.24 ± 0.04	1.21 ± 0.16	0.92 ± 0.14^c^	1.04 ± 0.08	0.95 ± 0.09	1.02 ± 0.19^c^	1.14 ± 0.16	1.08 ± 0.19
Number of Strokes	36 ± 10	30 ± 4	33 ± 3	38 ± 6	33 ± 5	39 ± 5^a^	36 ± 7^c^	32 ± 5	36 ± 5
Work/stroke (Nm)	20.3 ± 4.8	23.7 ± 4.8	21.4 ± 4.2	12.5 ± 1.8	13.6 ± 1.9	11.9 ± 1.5^a^	16.9 ± 4.8	18.9 ± 6.7	16.9 ± 5.7^a^
Peak Power (Watts)	53.6 ± 12.3	50.0 ± 5.7	50.5 ± 12.2	28.7 ± 6.5	33.1 ± 5.1	31.9 ± 5.3	41.1 ± 17.6	42.1 ± 11.6	41.8 ± 13.0
HR (bpm)	170 ± 15	166 ± 15	158 ± 18	168 ± 5	173 ± 6	158 ± 9^ab^	169 ± 12	169 ± 13	158 ± 13^ab^
La^-^ (mmol/L)	10.1 ± 3.7	9.9 ± 3.7	9.1 ± 2.0	9.6 ± 2.5	9.0 ± 1.5	8.0 ± 2.3^ab^	9.9 ± 3.3	10.0 ± 2.8	8.6 ± 2.3^ab^
RPE	16 ± 3	16 ± 2	17 ± 1	17 ± 2	16 ± 3	18 ± 1	17 ± 2	16 ± 2	17 ± 1

V_1_, post-season; V_2_, off-season; V_3_, pre-season.

*HR* Heart rate, *La* lactate, *RPE* Rating of perceived exertion.

^a^Significant difference between pre-season versus off-season (*p* < 0.05).

^b^Significant difference between pre-season versus post-season (*p* < 0.05).

^c^Significant difference between off-season versus post-season (*p* < 0.05).

Men exhibited poor sleep quality, as demonstrated by greater PSQI scores, in off-season compared to pre- and post-season (both *p* < 0.05; Table 2). There were 82.5% swimmers exhibited poor sleep quality across the entire season. Psychological parameters were not different for DALDA, WURSS-21, and AD-ACL scores across the three visits.

**Table 2:** Psychological responses to seasonal swim training across three visits.

	Men swimmers			Women swimmers			All		
	V_1_ (n = 8)	V_2_ (n = 6)	V_3_ (n = 8)	V_1_ (n = 7)	V_2_ (n = 4)	V_3_ (n = 7)	V_1_ (n = 15)	V_2_ (n = 10)	V_3_ (n = 15)
PSQI	8 ± 3	13 ± 4^ab^	6 ± 3	6 ± 2	9 ± 5	6 ± 4	7 ± 3	12 ± 5^ab^	6 ± 3
WURSS-21	19 ± 21	14 ± 13	15 ± 5	4 ± 5	6 ± 4	10 ± 10	12 ± 18	11 ± 11	13 ± 7
AD-ACL									
Energetic	12 ± 2	11 ± 3	10 ± 4	10 ± 4	9 ± 2	8 ± 2	11 ± 3	10 ± 2	9 ± 3
Tiredness	15 ± 3	15 ± 3	15 ± 4	15 ± 2	15 ± 2	15 ± 3	15 ± 3	15 ± 2	15 ± 3
Tension	10 ± 3	9 ± 2	9 ± 3	7 ± 2	6 ± 1	7 ± 3	9 ± 3	8 ± 2	8 ± 3
Calmness	13 ± 2	12 ± 2	12 ± 3	11 ± 3	11 ± 3	11 ± 2	12 ± 3	11 ± 2	11 ± 3
DALDA	8 ± 3	5 ± 3	8 ± 4	6 ± 7	5 ± 2	6 ± 4	7 ± 7	4 ± 2	7 ± 4

V_1_, post-season; V_2_, off-season; V_3_, pre-season.

*PSQI* Pittsburgh sleep quality index, *WURSS-21* Wisconsin upper respiratory symptom survey, *AD-ACL* Activation-deactivation adjective check list, *DALDA* Daily analysis of life demands of athletes.

^a^Significant difference between off-season versus pre-season (*p* < 0.05).

^b^Significant difference between off-season versus post-season (*p* < 0.05).

### Seasonal changes in physiological and psychological parameters

Table 3 shows the seasonal changes in physiological and psychological parameters. Swim speed significantly increased during the off-season phase compared to pre-season phase (*p* = 0.01) and in-season phase (*p* = 0.008). Swimmers had a significant decrease in HR during the pre-season phase compared to during off-season phase (*p* = 0.02) and in-season phase (*p* = 0.004). In pre-season phase, La^-^ was significantly decreased (*p* = 0.008) and RPE increased (*p* = 0.04) compared to in-season phase. Work/stroke was increased during off-season phase compared to pre-season phase (*p* = 0.04). PSQI scores during off-season phase was increased compared to other two phases (*p* = 0.001) and in-season phase increase the most when compared to of pre-season phase (*p* = 0.02). In addition, during pre-season phase, swimmer’s feeling of energy was significant decreased compared to the in-season phase (*p* = 0.04) and DALDA scores was increased than off-season phase (*p* = 0.04).

**Table 3:** Seasonal changes in physiological and psychological parameters.

	Off-season Phase	Pre-season Phase	In-season Phase	*p-*value
Speed (m/s)	0.09 ± 0.08 (6.8%)	− 0.07 ± 0.12 (− 5.9%)^a^	− 0.06 ± 0.17 (− 5.5%)^c^	0.01
HR (bpm)	3 ± 7 (1.3%)	− 6 ± 10 (− 3.4%)^ab^	5 ± 8 (3.2%)	0.01
La^− ^ (mmol/L)	0.6 ± 1.8 (6.2%)	− 1.1 ± 1.6 (− 11.9%)^b^	1.2 ± 2.3 (13.9%)	0.03
RPE	0 ± 2 (0%)	1 ± 2 (6.3%)^b^	− 1 ± 2 (− 5.8%)	0.11
Number of Strokes	− 1.1 ± 3.4 (− 2.7%)	3.8 ± 4.1 (12.3%)	0.6 ± 6.7 (1.7%)	0.15
Work/stroke (Nm)	0.6 ± 1.6 (2.1%)	− 2.3 ± 3.0 (− 11.6%)^a^	− 0.3 ± 3.1 (− 1.8%)	0.09
Peak Power (Watts)	− 1.9 ± 11.2 (− 2.2%)	− 0.6 ± 7.9 (− 1.4%)	0.4 ± 7.0 (0.9%)	0.85
PSQI	4.4 ± 3.9 (63.9%)	− 6.2 ± 4.4 (− 54.4%)^ab^	0.6 ± 3.7 (9.4%)^c^	0.001
WURSS-21	− 1.1 ± 18.5 (− 9.2%)	4.4 ± 12.3 (39.6%)	− 0.4 ± 19.2 (− 3.2%)	0.73
AD-ACL (Energetic)	− 0.2 ± 3.3 (− 1.8%)	− 1.3 ± 3.3 (− 12.9%)^b^	1.7 ± 3.3 (18.7%)	0.09
AD-ACL (Tiredness)	0.2 ± 2.8 (1.3%)	− 0.5 ± 4.1 (− 3.3%)	− 0.2 ± 4.6 (− 1.3%)	0.93
AD-ACL (Tension)	− 1.4 ± 3.3 (− 15.6%)	0.3 ± 2.8 (4.1%)	0.7 ± 3.1 (8.6%)	0.24
AD-ACL (Calmness)	− 0.5 ± 3.5 (− 4.2%)	0.1 ± 3.2 (0.9%)	0.6 ± 2.4 (5.3%)	0.67
DALDA	− 3.1 ± 7.7 (− 44.3%)	3.7 ± 4.9 (100%)^a^	− 0.3 ± 8.0 (− 4.2%)	0.12

Data represented as Mean ± SD (percent change) Off-season Phase, V_2_–V_1_; Pre-season Phase, V_3_–V_2_; In-season Phase, V_1_–V_3_. V_1_, post-season; V_2_, off-season; V_3_, pre-season.

*HR* Heart rate, *La*^-^ lactate, *RPE* Rating of perceived exertion, *PSQI* Pittsburgh sleep quality index, *WURSS-21* Wisconsin upper respiratory symptom survey, *AD-ACL* Activation-deactivation adjective check list, *DALDA* Daily analysis of life demands of athletes.

^a^Significant difference between pre-season phase and off-season phase (*p* < 0.05).

^b^Significant difference between pre-season phase and in-season phase (*p* < 0.05).

^c^Significant difference between in-season phase and off-season phase (*p* < 0.05).

### Relationships between physiological and psychological parameters

**Figure 3:**
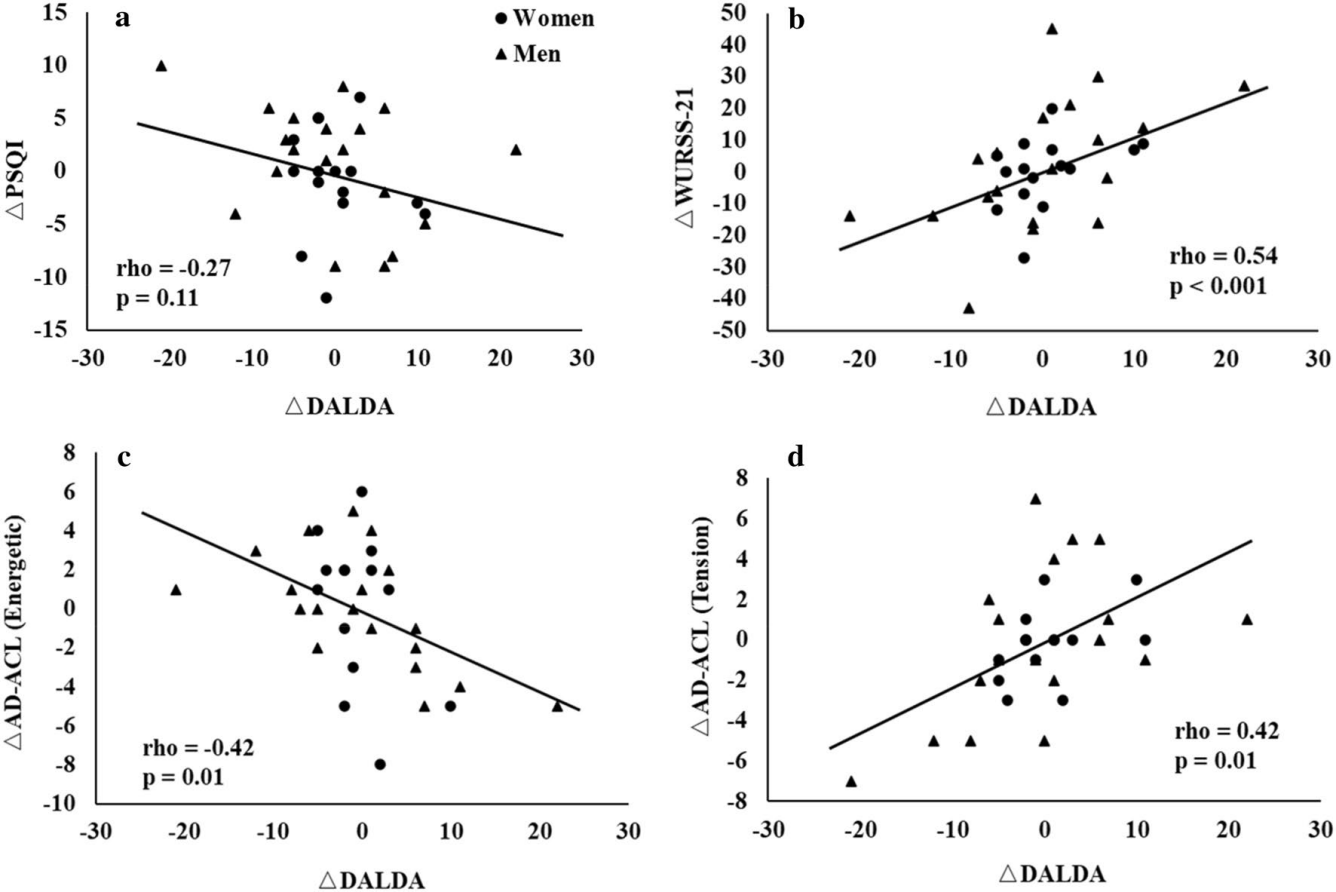
The correlations between ΔDALDA and ΔPSQI (**a**), ΔWURSS-21 (**b**), and ΔAD-ACL (Energetic: (**c**) and Tension: (**d**)). DALDA: Daily Analysis of Life Demands of Athletes; PSQI: Pittsburgh Sleep Quality Index. WURSS-21: Wisconsin Upper Respiratory Symptom Survey; DALDA: Daily Analysis of Life Demands of Athletes. AD-ACL: Activation-Deactivation Adjective Check List.

**Figure 4:**
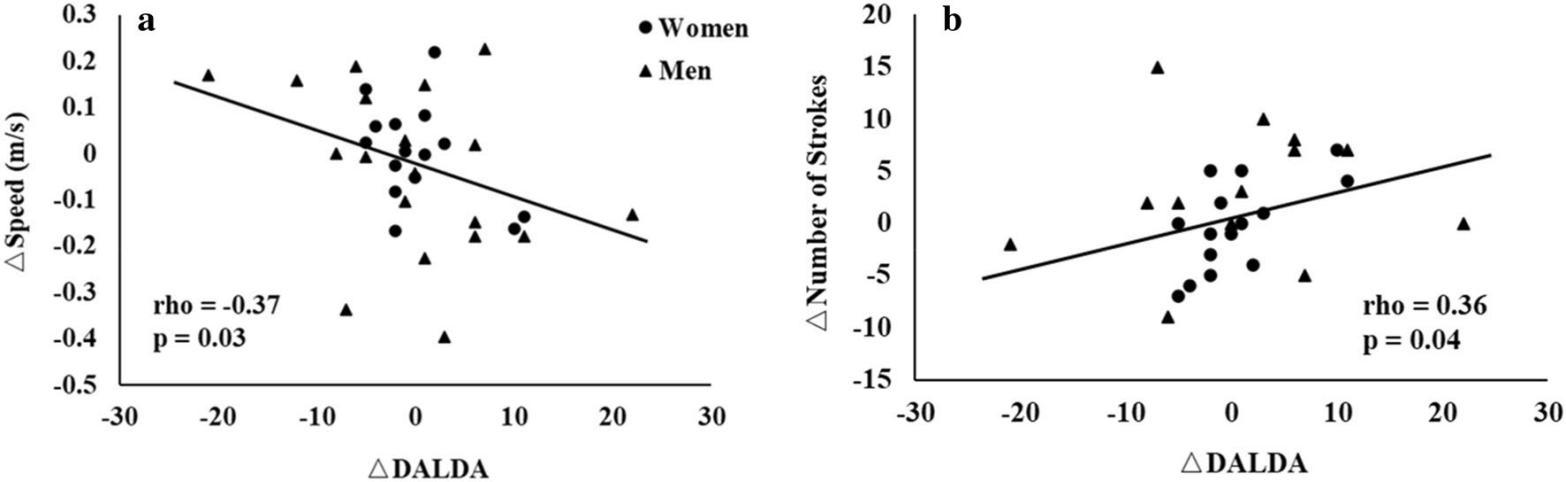
The correlations between Δ DALDA and speed (**a**) and Δ number of strokes (**b**). DALDA: Daily Analysis of Life Demands of Athletes; Swimming speed and number of strokes data were selected at submaximal workload (men: 36 kg and women: 23 kg).

Table 4 shows the correlation matrix of the changes in psychological, physiological parameters, and swim performance. Among the psychological assessments, no correlation was found between the change in DALDA scores and PSQI scores (*rho* = − 0.27, *p* = 0.11, Fig. 3a). An increase in DALDA scores was associated with an increase in WURSS-21 score (*rho* = 0.54, *p* < 0.001, Fig. 3b), being less energetic (*rho* = − 0.42, *p* = 0.01, Fig. 3c), and more tension state (*rho* = 0.42, *p* = 0.01, Fig. 3d) from AD-ACL questionnaire. In addition, an increase in DALDA scores were significantly related to a decrease in speed (*rho* = − 0.37, *p* = 0.03; Fig. 4a) and an increase in the number of strokes (*rho* = 0.36, *p* = 0.04; Fig. 4b) at submaximal tethered workload. An increase in RPE was significantly associated with increase in WURSS-21 score (rho = 0.36, *p* = 0.04) and being less energetic state (rho = − 0.34, *p* = 0.04). An increased in peak power was significantly associated with an increase in the scores of WURSS-21 (rho = 0.46, *p* = 0.01) and DALDA (rho = 0.38, *p* = 0.04), but not related to the change in swim speed (rho = 0.07, *p* = 0.72). However, an increase in work/stroke was significantly associated an increase in swim speed (rho = 0.82, *p* < 0.001) and a decrease in RPE (rho = − 0.63, *p* < 0.001).

**Table 4:** Correlation matrix of the changes in psychological, physiological parameters, and swim performance.

	PSQI	WURSS-21	Energetic	Tiredness	Tension	Calmness	DALDA	Speed	HR	La^-^	RPE	Peak Power
PSQI	1											
WURSS-21	− 0.13	1										
Energetic	0.05	− 0.06	1									
Tiredness	0.18	− 0.39*	− 0.45*	1								
Tension	− 0.03	0.22	0.05	0.03	1							
Calmness	− 0.07	0.25	− 0.31	0.16	0.02	1						
DALDA	− 0.27	0.54*	− 0.42*	0.01	0.42*	0.32	1					
Speed	0.18	− 0.34*	0.03	0.21	− 0.35*	− 0.27	− 0.37*	1				
HR	0.16	− 0.14	0.37*	− 0.17	− 0.02	− 0.07	0.05	0.09	1			
La^− ^	0.30	0.04	− 0.14	0.23	0.13	0.21	− 0.04	− 0.23	0.44*	1		
RPE	− 0.09	0.36*	− 0.34*	− 0.28	0.18	0.14	0.26	− 0.42*	0.01	0.27	1	
Peak power	− 0.13	0.46*	− 0.07	− 0.11	0.26	0.15	0.38*	0.07	− 0.05	− 0.13	0.03	1
Work/Stroke	0.29	− 0.32	− 0.02	0.35	− 0.19	− 0.20	− 0.28	0.82*	0.19	− 0.06	− 0.63*	0.23

*DALDA* Daily analysis of life demands of athletes, *PSQI* Pittsburgh sleep quality index, *WURSS-21* Wisconsin upper respiratory symptom survey, *DALDA* Daily analysis of life demands of athletes, *HR* Heart rate, *La* lactate, *RPE* Rating of perceived exertion.

*Significant correlations between outcome variables (*p* < 0.05).

Significant values are in [bold].

## Discussion

The present study aimed to examine physiological and psychological parameters among collegiate swimmers on three occasions during an academic year. A better swim performance was observed during off-season compared to pre-season and post-season across all athletes. Women had the lowest HR and lactate concentration at pre-season compared to other timepoints. In addition, an increased in stress sources and symptoms assessed by DALDA was associated with an increased in upper respiratory illness, being less energetic and greater tension state, greater number of strokes, and a decreased in swim speed.

Contrary to our hypothesis, swimmers seemed to have better exercise performance, to some extent, at the end of the off-season timepoint compared to the pre- and post-season. Swim speed was faster and swimmers used fewer strokes exhibiting greater work per stroke in the tethered anaerobic test after the off-season. There was a trend showing that men swimmers performed 0.13 m/s faster in 6 fewer strokes and greater work per stroke to finish the tethered test at off-season compared to post-season. Women particularly exhibited a trend or significant 0.9–0.12 m/s faster swim speed during off-season compared to other timepoints, as a result of fewer stroke number and greater work per stroke. The present results suggest during off-season swimmers have greater stroke efficiency resulted in a fast speed in this anaerobic tethered test. In the present study, swimmers had a weekly target swim volume of 28.6 km for sprinters and 36.6 km for mid-distance swimmers. At post-season immediately after the NCAA championships, swimmers in the present study may have lacked recovery time in accordance with stress from intensive training stimulus. This cohort’s swim volume was comparable to previous research suggesting a greater swim volume significantly impacted muscular soreness possibly altering shoulder function and stroke mechanics, which are the major factors for success in swimming^19,20^.

Women had 10 ~ 15 bpm lower HR and 1.0 ~ 1.6 mmol/L lower La^-^with similar RPE given the same resistance workload at pre-season compared to the other two timepoints. This result is consistent to our hypothesis suggesting physiological adaptations occurred with an increased training volume that reduced cardiovascular stress for a given perceived exertion. However, anaerobic tethered swim performance was not improved as physiological adaptation occurred at pre-season. Yet, swim performance in pre-season was 2.1 s slower than off-season and similar to post-season. Although not significant, nine hundredths of a second may be a meaningful difference between competitors in a swim race. At the Olympic level, improving swim performance by 2.9 s may substantially improve an athletes’ chance to medal^21^. Clemente-Suarez et al. determined physiological and psychological features associated with anaerobic and aerobic swim performance, psychological parameters were related to anaerobic swimming effort, whereas physiological parameters (HR, La^-^) were more related to aerobic than anaerobic swim performance^10^. In the present study, swimmers may have felt less pressure and/or distraction from academics and the demands on pre-season and post-season training, making the off-season anaerobic testing enjoyable and uniquely competitive.

Sleep quality is essentially important for athletes and is negatively associated with athletic performance and health. Training schedules, practice times, travel to competitions, and pre-competition anxiety are the factors impacting an athlete’s sleep quality and/or quantity^22^. In the present study, swimmers reported poor sleep quality according to high PSQI score throughout the entire season. Surda et al. suggested that perceived sleep quality is more likely associated with hours of sleep in the night, and sleep hours typically was shortened in swimmers prior to the early morning training session^23^. In the present study, swimmers typically trained in the early morning session (6:00 AM) 5 days/wk. Even in off-season, swimmers still had the same amount of morning training sessions and similar swim volumes compared to post-season. A previous study showed reduced sleep quantity has a direct physiological effect inducing impaired athletic performance, reduced glycogen stores, increased heart rate, and elevated lactate concentration^24^. However, the present study did not show subjective sleep quality was linked to swim performance. Swimmers probably have overestimated their PSQI score as a result of psychological compensation, where they did not have enough sleep hours that they believed they should have during off-season. Thus, the current data showed that PSQI questionnaire may not actually suggest a swimmers’ sleep quality differences between seasons^23^.

The present study did not observe any differences in DALDA, WURSS-21, and AD-ACL scores across the visits. Swimmers’ self-reported upper respiratory illness was similar throughout the season, suggesting swim volume elevation did not significant impact their immune functions. This result contradicts the likely outdated “open window” theory, which purports that immune function is suppressed following endurance exercise, thus leading to an increased susceptibility to upper respiratory illness^25^. This theory has however been challenged recently by Campbell et al.^26,27^ with studies failing to identify pathogenic causes in the majority of athletes exhibiting upper respiratory tract infection symptoms. Our results further advocates for the inadequacy of the open-window theory, since the swimmers recruited in this study had similar illness and perceived well-being throughout the season. While this suggests that they were either equally sick or not sick throughout all timepoints, a clear conclusion cannot be drawn from our results since swim is a year-round sport, and thus a true baseline assessment could not be collected. Establishing a consistent baseline visit for psychological assessments may allow for a greater sensitivity to changes throughout a season^28^. On the other hand, AD-ACL is a state measure, which captures psychological constructs in the moment. In other words, mood state could be changed every few minutes and AD-ACL may not reflect a swimmer’s mood state from a chronic accumulated stress as comparing the differences between the visits. However, considering the ease of survey administration, it could be argued that coaches and strength conditioning teams would benefit in routinely using surveys to track their athletes’ well-being as an additional consultation tool, without over-interpreting them.

As considering psychological and physiological responses to training stimulus are more likely an individual process. Uniquely, the present study analyzed the dynamic changes in physiological and psychological outcomes during the pre-season phase, off-season phase, and in-season phase throughout the year. Swim speed increased (6.8%) only during off-season phase compared to other two phases where swim speed declined. Theall et al. estimated seasonal stress load in college swimmers by using self-report questionnaire data combined with known academic, training, and competition burden creating a composite score^29^. It showed that high stress was observed during post-season, where championships overlap with academic exam schedules. Low stress was observed during the off-season and moderate stress was seen during pre-season where swimmers are ramping into a new competition season with building academic work. Life event stress sources and symptoms in collegiate athlete stem from both training (excessive training volume or inadequate rest) and non-training stimuli (emotional, social, and academic)^30^. Thus, swimmers might have exhibited lower stress level while swim performance is not competing in off-season than during the competitive season. During the pre-season phase, swim speed (-5.9%) and work/stroke (-11.6%) decreased with a greatest elevation in DALDA scores (100%) when compared to off-season phase. Previous study showed using DALDA questionnaire can distinguish different training periods among rest, training days, and pre- and post-match days, with greater DALDA scores observed on training days compared to rest and match days^31^. Although physiological adaptations may occur according to the change in La^-^ and HR but swimmers possibly were already experiencing high psychological stress as they felt more physical effort and less energetic under the same workload. These data support the aforementioned findings, where swimmers progressed into the competitive season with an increased training volume, they likely experienced more psychological stress, and consequently impacted anaerobic swim performance.

The present study also found an increase in stress sources and symptoms from the DALDA was related to an increased in upper respiratory infection rate, being less energetic and greater tension state. In agreement with Moreira et al. where the upper respiratory infection rate (WURSS-21) and DALDA scores were correlated, as a result of one week high training load^32^. Swimmers who had greater stress sources and symptoms were in a low energy and tended to have a high-tension state. The Activation-Deactivation Adjective Check List (AD-ACL) measures a psychological syndrome expressed the feeling and arousal, especially for an individual sport^33^. Psychological assessment is necessary to investigate any threat to well-being of a college student, in terms of early prevention of overtraining and/or “staleness” in swim. In addition, an increase in DALDA scores were also associated with a greater decrease in swim speed and increase in the number of swim strokes, suggesting swimmers who elevate stress sources and symptoms has a negatively impact on swim performance. The majority of previous research has investigated the relationships between psychological and physiological stress but not exercise performance in swimming^31–33^. In the present study, a year-round training program was found to alter physiological adaptation and psychological stress, potentially impacting swim performance. Interestingly, the present study found an increased in peak power was associated with an increased in upper respiratory infection rate and DALDA scores, but not correlated with work per stroke or swim speed. In swim events, successful swimmers not only need to generate greater power, but also need an efficient stroke for propulsive force production as a result of fast speed in the pool. The present results may suggest swimmers who are undergoing greater stress sources and symptoms were struggled to pull the resistance in the anaerobic power test, consequently altered swim mechanics generating an inefficient stroke in any second^34^.

### Practical application

Monitoring the health of swimmers should include a combination of physiological and psychological markers to better understand the athletes as a whole. Markers of stress and poor performance peaked just after the post-competition, potentially in response to the psychological and physical intensity of the season, overtraining, or lack of recovery activities. This study demonstrated subtle changes in subjective measures relate swim performance and a true baseline may be necessary to detect more sensitive changes across the season. However, one limitation was a small sample size, thereby limiting out ability to further identify sex and swim event effects (sprinters vs. mid-distance swimmers) on the main outcomes of physiological and psychological parameters. Also, some dropped cases occurred (6 men and 4 women swimmers) during off-season resulted in unequal sample size across three visits. In order to confirm our assumptions from the mixed model, we limited our analysis to only those swimmers with completed data sets. Consequently, the results were similar across all metrics. In athletic research, it is not always feasible to conduct large sample, population level studies. On the other hand, small sample studies provide very rich information about sample-specific phenomena in a longitudinal study design^35^. The present results will provide key information for the future studies in other swim teams (i.e. effect size estimates). While coaches and practitioners should cautiously interpret these assessments individually, take together the tethered swim test and questionnaires may be beneficial in directing swimmers to peak their performance when needed.

## Conclusion

This is the first study showing that increased DALDA scores are related to impaired swim performance in a year-round, ecologically-relevant setting. Thus, coaches and athletes should consider monitoring psychological and physiological parameters in their training to achieve and maintain a high swim performance. The present study suggests subjective psychological questionnaires alongside the anaerobic tethered swim performance assessments can be useful tools to identity the chance of overreaching/overtraining in swimmers. The present data reinforce that physiological and psychological aspects should be considered in accordance with exercise performance and training volume when preparing swimmers for competition.

## Data Availability

The data support the findings of the present study that are available from the corresponding author upon reasonable request.
